# RSPH14 regulates the proliferation, cell cycle progression, and apoptosis of non‐small cell lung cancer cells

**DOI:** 10.1002/2211-5463.13266

**Published:** 2021-08-25

**Authors:** Ke Ma, Jun Peng, Hao Rong, Yanhua Jiang, Huachuan Zhang, Jiang Zhu, Bo Xiao, Peng Tang, Jintao He, Zhentao Yu

**Affiliations:** ^1^ Department of Esophageal Cancer Tianjin Medical University Cancer Institute and Hospital Key Laboratory of Cancer Prevention and Therapy of Tianjin Tianjin’s Clinical Research Center for Cancer National Clinical Research Center of Cancer Tianjin China; ^2^ Department of Thoracic Surgery Sichuan Cancer Hospital & Institute Sichuan Cancer Center, School of Medicine University of Electronic Science and Technology of China Chengdu China

**Keywords:** apoptosis, RSPH14, Non‐small cell lung cancer, proliferation, cell cycle, biomarker

## Abstract

Non‐small cell lung cancer (NSCLC) is the most common subtype of lung cancer, and it is characterized by a high incidence. It is important to understand the molecular mechanisms that determine the progression and metastasis of NSCLC in order to develop more effective therapies and identify novel diagnostic indicators of NSCLC. RSPH14 has been reported to be related to multiple human diseases, including duodenal adenocarcinoma and meningiomas, but the role of RSPH14 in NSCLC remains unclear. The present study aimed to investigate the molecular function and clinical significance of RSPH14 in NSCLC. Analyses of public datasets and clinical samples demonstrated that RSPH14 expression was upregulated in NSCLC samples compared with normal samples. In addition, high RSPH14 expression was associated with a shorter overall survival time in patients with NSCLC. Notably, RSPH14 knockdown suppressed the proliferation and cell cycle progression and enhanced the apoptosis of NSCLC cells. Mechanically, tandem mass tag analysis demonstrated that RSPH14 can affect multiple processes, including the AMPK signaling pathway, calcium ion import regulation, glucose transmembrane transporter activity, and glucose transmembrane transport. Taken together, the results of the present study suggest that RSPH14 may be a promising prognostic factor and therapeutic target for NSCLC.

## Graphical abstract

RSPH14 expression was upregulated in NSCLC samples compared with normal samples and was involved in regulating multiple processes, including glucose transmembrane transport and the AMPK signaling pathway. High expression of RSPH14 enhanced proliferation and cell cycle progression and suppressed the apoptosis of NSCLC cells, thereby promoting the progression of NSCLC.

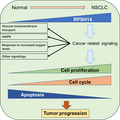

**Abbreviations**
NSCLC: non‐small cell lung cancer
SCLC: small cell lung cancer
lncRNAs: long noncoding RNAs
shRNA: short hairpin RNA
CCK‐8: cell counting Kit‐8
PI: propidium iodide
KEGG: kyoto encyclopedia of genes and genomes

Histologically, lung cancer is classified into small cell lung cancer (SCLC) and non‐small cell lung cancer (NSCLC) [1]. NSCLC is the most common subtype of lung cancer, accounting for 80% of all cases (1,2). However, NSCLC is more resistant to chemotherapy compared with SCLC [2]. Over the past decades, the incidence and mortality rates of NSCLC have increased rapidly. In addition, the 5‐year survival rate of NSCLC remains low compared with other common cancers, such as colorectal, breast, and prostate cancers [2]. The 5‐year survival rates for patients with advanced and metastatic NSCLC are 24 and 4%, respectively [3]. Previous studies have reported a series of regulators involved in controlling NSCLC metastasis, including TWIST1 [4], PTCH1 [5], and TGF‐β‐induced long noncoding RNAs (lncRNAs) [6]. Identification of further biomarkers is urgently required to improve the prognosis and survival of patients with NSCLC.

Cancer is caused by the abnormal regulation of cell cycle progression and apoptosis [7]. Apoptosis is affected by intracellular and/or extracellular signals and is characterized by cell morphological changes that target death, including nuclear division and concentration, mitochondrial outer membrane permeability, membrane hemorrhage, cell atrophy, and apoptotic body formation [8]. Recently, activation of apoptosis has been proven to be a therapeutic biomarker for cancer by decreasing the accumulation of cancer cells [8]. In NSCLC, several novel apoptosis regulators have been identified, including MINCR [9, 10] and LINC00961 [11]. MINCR is a cell cycle and apoptosis regulator in NSCLC [9, 10], while lncRNA LINC00961 suppresses tumor growth and promotes cell apoptosis in NSCLC [11]. However, the molecular mechanisms underlying the regulation of NSCLC remain unclear.

RSPH14 is a new gene with a slight homology to a yeast VAC8 protein, which transports aminopeptidase I from the cytoplasm to the vacuole in vacuole inheritance [12]. RSPH14 has been reported to be heterozygously or homozygously lost in pediatric rhabdoid tumors [13]. Recently, several studies also demonstrated this gene was related to multiple human diseases, such as duodenal adenocarcinoma [14], congenital heart disease [15], meningiomas [16], and circulating parathyroid hormone formation [17]. However, the role of RSPH14 in NSCLC remains unclear. The present study aimed to investigate the function and prognostic value of RSPH14 in NSCLC cells. The function of RSPH14 in inducing the proliferation and apoptosis of NSCLC cells and the role of the AMPK signaling pathway in this process were investigated, in order to determine whether RSPH14 may serve as a promising biomarker for the treatment of patients with NSCLC.

## Materials and methods

### Patients and tissue samples

A total of nine NSCLC tissue samples were collected from patients with NSCLC at Si Chuan Cancer Hospital between 2016 and 2018, according to postoperative pathological reports. The present study was approved by the Ethics Committee of the Si Chuan Cancer Hospital, and written informed consent was provided by all patients prior to the study initiation. The use of human samples complies with the standards stipulated in the Declaration of Helsinki.

### Bioinformatics analysis

The Kaplan–Meier Plotter database (http://www.kmplot.com) was used to determine the prognostic value of RSPH14 in NSCLC. The Database for Annotation, Visualization and Integrated Discovery (version 6.7; http://david.abcc.ncifcrf.gov) was used to perform functional analysis [18].

### Lentiviral constructs and transfection

A recombinant lentiviral vector carrying RSPH14 short hairpin (sh)RNA was constructed using standard molecular techniques. The expression of RSPH14 shRNA in infected cells was verified via reverse transcription‐quantitative (RT‐q)PCR analysis. The RSPH14 shRNA sequence was as follows: CCGGGATCATCAGCAAAGGTCTGATCTCGAG ATCAGACCTTTGCTGATGATCTTTTTG. An empty vector was used as control shRNA. The control shRNA sequence was as follows: CCGGTTCTCCGAACGTGTCACGTCTCGAGACGTGACACGTTCGGAGAATTTTTG.

### Cell culture

NCI‐H1975, A549, and H1299 cells were purchased from the American Type Culture Collection and maintained in RPMI‐1640 medium supplemented with 10% fetal bovine serum (HyClone; Cytiva), at 37 °C in 5% CO_2_.

### RT‐qPCR

Total RNA was extracted from cells using TRIzol® reagent (Invitrogen; Thermo Fisher Scientific, Waltham, MA, USA) and reverse‐transcribed into cDNA using the PrimeScript™ RT Reagent kit (Takara Biotechnology, Shiga, Japan). qPCR was subsequently performed using the SYBR^®^ Green qPCR kit (Takara Biotechnology), on the ABI StepOnePlus real‐time PCR system (Applied Biosystems; Thermo Fisher Scientific). The following thermocycling conditions were used for qPCR: initial denaturation at 95 °C for 30 s; followed by 40 cycles at 95 °C for 5 s and 60 °C for 34 s, according to the manufacturer’s protocol. The following primer sequences were used for PCR: RSPH14 forward, 5’‐AGAAGAACGAATGGTTGAGAT‐3’ and reverse, 5’‐TCCTGGATATTTGCAGTCAGT‐3’; and GAPDH forward, 5’‐TGACTTCAACAGCGACACCCA‐3’ and reverse, 5’‐ CACCCTGTTGCTGTAGCCAAA‐3’. Relative expression levels were calculated using the 2^−ΔΔCq^ method and normalized to the internal reference gene GAPDH.

### Plate analysis with the adherent cell cytometry system Celigo®

Briefly, cells were stained with fluorescent nuclear stain (Hoechst nuclear stain, 2.6 μg·mL^−1^, Invitrogen; Thermo Fisher Scientific). As previously described [18], the adherent cell cytometry system was used to quantify the fluorescence expression of cells. Use equipped with bright field and fluorescence channels adherent cells cytometry analysis plate: Hoechst blue (nuclear staining of DAPI) filter and siGLO green filter. The gating parameters of each fluorescence channel were adjusted to exclude background noise and other nonspecific signals. Quantitative analysis for each fluorescence channel and individual well was performed using the Celigo® system, including the total count of gated events and the average integrated red fluorescence intensity.

### Cell proliferation assay

The Cell Counting Kit‐8 (CCK‐8) assay was performed to assess cell viability following infection with shRSPH14. A549 and H1299 cells were seeded into 96‐well plates at a density of 5000 cells·well^−1^. Cell viability was assessed at days 0–4, according to the manufacturer’s protocol. Following incubation with CCK‐8 reagent for 4 h, cell viability was analyzed at a wavelength of 490 nm using a PowerWave XS microplate reader (BioTek Instruments, Winooski, VT, USA). All experiments were performed in triplicate.

### Adherent cell cytometry system Celigo® assay

Cells infected with shRSPH14 or control shRNA were trypsinized and seeded into 96‐well plates at a density of 2000 cells·well^−1^. Fluorescence was measured every day for a total of 5 days using the Celigo® system, as previously described.

### Cell cycle assay

Cells infected with shRSPH14 or control shRNA were harvested and washed three times with PBS. Cells were subsequently incubated with PBS containing 0.03% Triton X‐100, 100 ng·mL^−1^ RNase A, and 50 ng·mL^−1^ propidium iodide (PI) for 15 min. Cell cycle distribution was analyzed using a FACSanto flow cytometer (BD Biosciences, San Jose, CA, USA) and modfit LT 3.0 software. All experiments were performed in triplicate.

### Cell apoptosis assay

Cells infected with shRSPH14 or control shRNA were harvested and washed three times with PBS. Cells were subsequently treated with 1X binding buffer and stained with 10 µL Annexin V‐APC staining solution (eBioscience; Thermo Fisher Scientific). Apoptotic cells were subsequently analyzed using a FACSanto flow cytometer (BD Biosciences) and modfit lt 3.0 software. All experiments were performed in triplicate.

### Western blotting

Total protein was extracted from cells using RIPA buffer (Thermo Fisher Scientific). Total protein was quantified using the Pierce BCA protein detection kit (Thermo Fisher Scientific) and 20 µg of protein/lane was separated by 10% SDS/PAGE. The separated proteins were subsequently transferred onto PVDF membranes (EMD Millipore) and blocked with TBST with 5% milk at room temperature for 2–4 h. The membranes were incubated with primary antibodies against RSPH14 (1:1000; cat. no. A15430; ABclonal Biotech Co., Boston, MA, USA) and GAPDH (1:1000; cat. no. sc‐32233; Santa Cruz Biotechnology, Santa Cruz, CA, USA) overnight at 4 °C. Following the primary incubation, membranes were incubated with horseradish peroxidase‐conjugated goat antimouse IgG secondary antibodies (1:5000; cat. no. abs20001ss; ABSIN) for 2 h at 37^−1^°C. Protein bands were visualized using enhanced chemiluminescence (Bio‐Rad Laboratories, Inc.) and analyzed using graphpad prism 6.0 software (GraphPad Software Inc., La Jolla, CA, USA).

### Statistical analysis

Statistical analysis was performed using graphpad 6.0 software (GraphPad Software Inc.). Data are presented as the mean ± standard deviation. Student’s *t*‐test or Mann–Whitney *U*‐test were used to compare differences between groups. Survival analysis was performed using the Kaplan–Meier method and log‐rank test. The association between RSPH14 expression and cancer prognosis was assessed. *P* < 0.05 was considered to indicate a statistically significant difference.

## Results

### Expression of RSPH14 in NSCLC

**Fig. 1:**
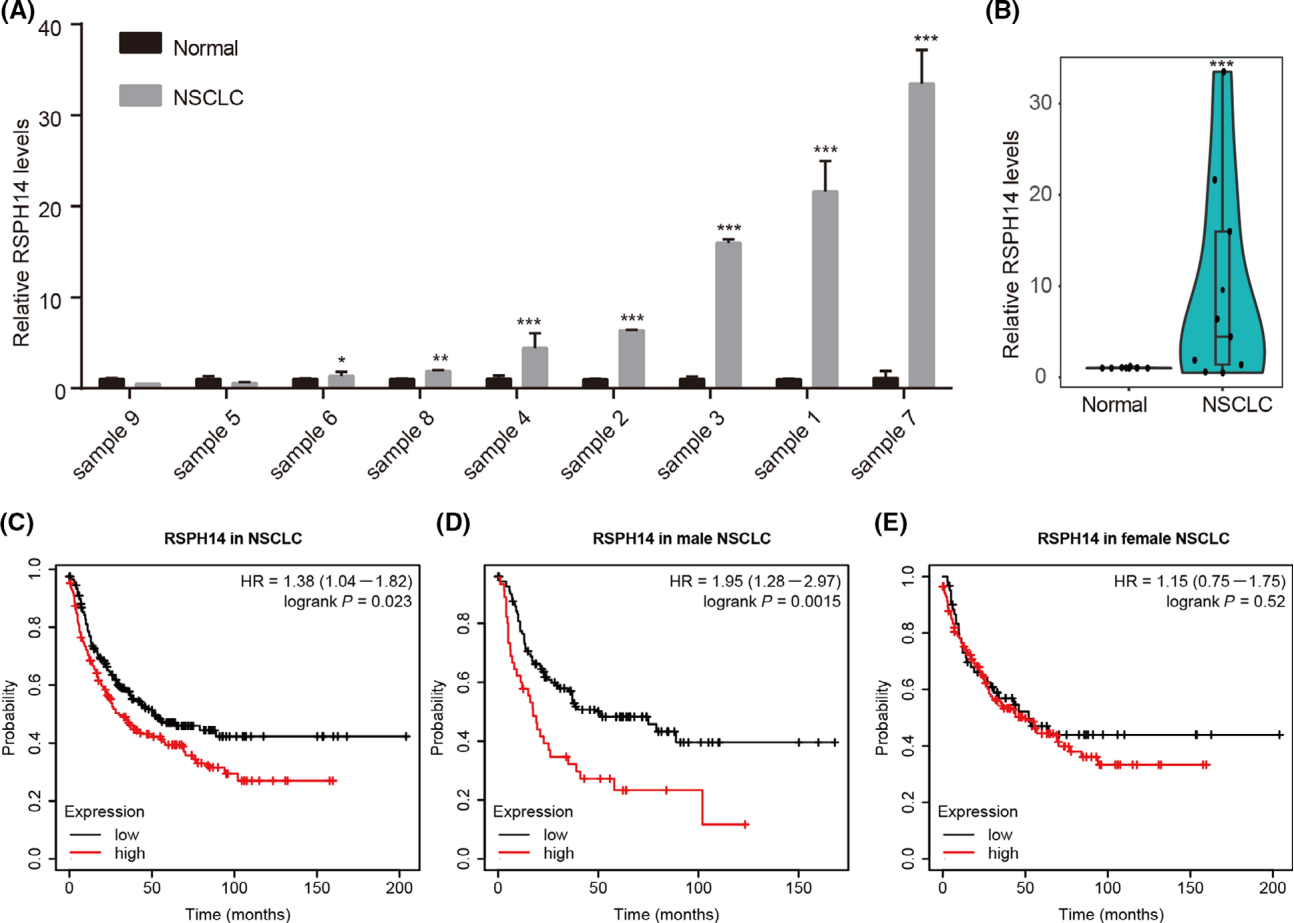
The clinical significance of RSPH14 expression in NSCLC tissue. (A–B) The RSPH14 expression in NSCLC tissues (*n* = 9) was remarkably higher than in that matched normal samples (*n* = 9). RSPH14 expression was detected using qRT‐PCR. Student’s t‐test was used to compare differences between groups. *P < 0.05, **P < 0.01, ***P < 0.001 vs. nontumor control. (C–E) Kaplan–Meier curve analysis showed higher RSPH14 expression correlates to shorter OS time in NSCLC (C), male NSCLC (D), and female NSCLC (E) by analyzing Kaplan–Meier plotter datasets. All data are shown as mean ± SD of three independent experiments.

To determine whether RSPH14 is involved in the regulation of NSCLC progression, RSPH14 mRNA expression was detected in NSCLC tissues and corresponding normal tissues. The results demonstrated that RSPH14 mRNA expression was upregulated in NSCLC tissues compared with normal tissues (Fig. 1A and B). The specific expression characterizations of RSPH14 indicated that this gene may be associated with the progression of NSCLC.

### RSPH14 is associated with poor prognosis in NSCLC

The characteristics of patients with high and low RSPH14 mRNA expression levels were compared. Patients were divided into two groups (high and low expression groups), and the cut‐off was determined using the Kaplan–Meier plotter database. Analyses of the Kaplan–Meier plotter datasets demonstrated that lower RSPH14 mRNA expression levels had a longer overall survival time than those with high RSPH14 mRNA expression levels in patients with NSCLC (Fig. 1C) and male patients with NSCLC (Fig. 1D). However, we did not observe a significant correlation between RSPH14 expression and OS in female patients with NSCLC (Fig. 1E).

### RSPH14 expression is upregulated in NSCLC cells and is associated with cell proliferation

**Fig. 2:**
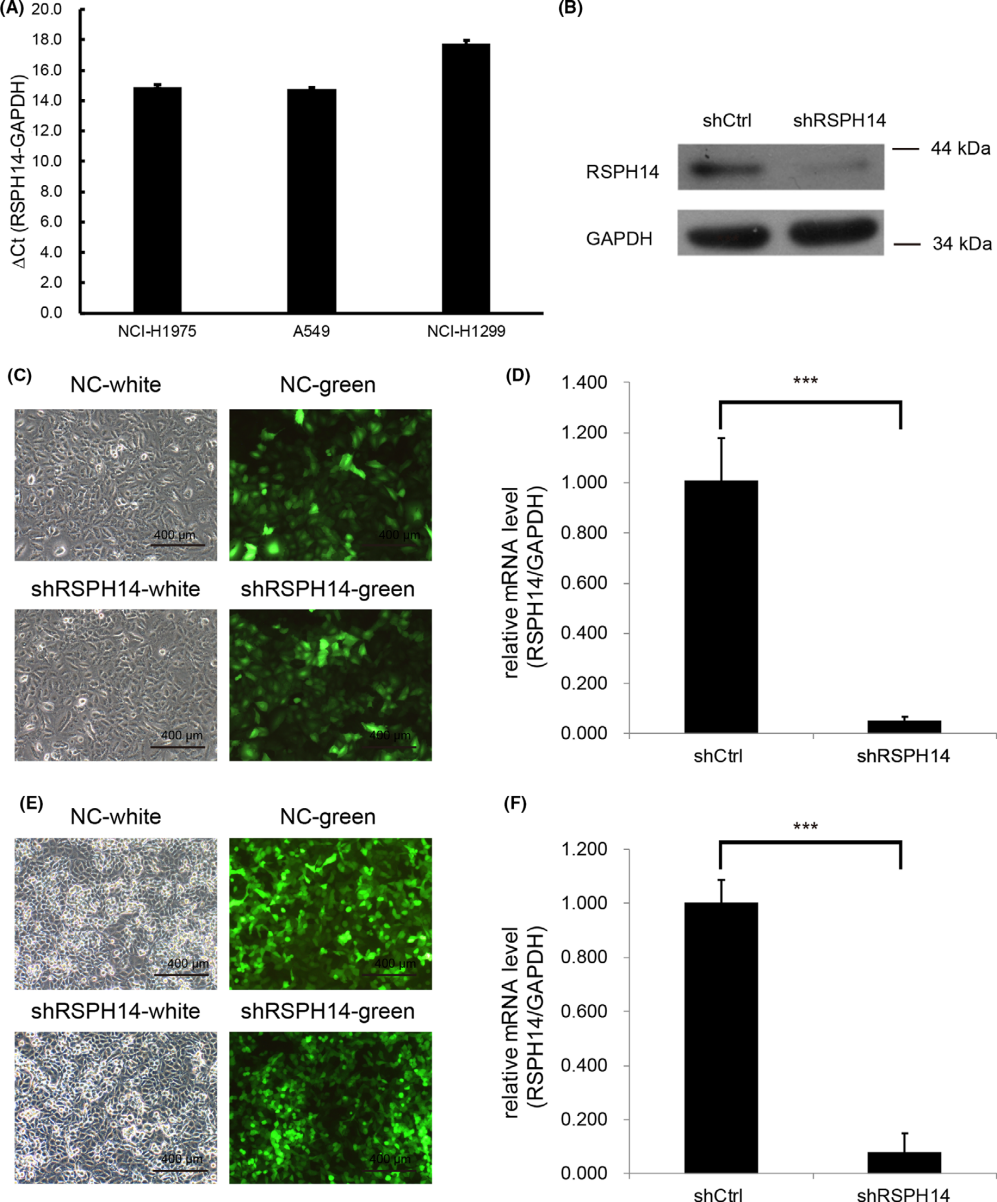
The suppressing effects of downregulated RSPH14 in NSCLC cell with shRSPH14 transfection. (A) The expression levels of RSPH14 were detected in 3 CRC cell lines including H1975, A549, and H1299 cells by using RT‐qPCR assay. (B) The downregulation of RSPH14 in protein levels with lentivirus‐mediated shRSPH14 in A549 cells by using western blot assay with primary antibodies against RSPH14 (1:1000; cat. no. A15430; ABclonal Biotech Co.) and GAPDH (1:1000; cat. no. sc‐32233; Santa Cruz Biotechnology). (C) The infection efficiency in A549 cells was detected by fluorescence imaging. Magnification ×100. (D) The downregulation of RSPH14 in mRNA levels with lentivirus‐mediated shRSPH14 in A549 cells by using RT‐qPCR assay. (E) The infection efficiency in H1299 cells was detected by fluorescence imaging. Magnification ×100. (F) The downregulation of RSPH14 in mRNA levels with lentivirus‐mediated shRSPH14 in H1299 cells by using RT‐qPCR assay. Scale bars: 400 μm. Student’s t‐test was used to compare differences between groups. ***P < 0.001 vs. control. All data are shown as mean ± SD of three independent experiments

**Fig. 3:**
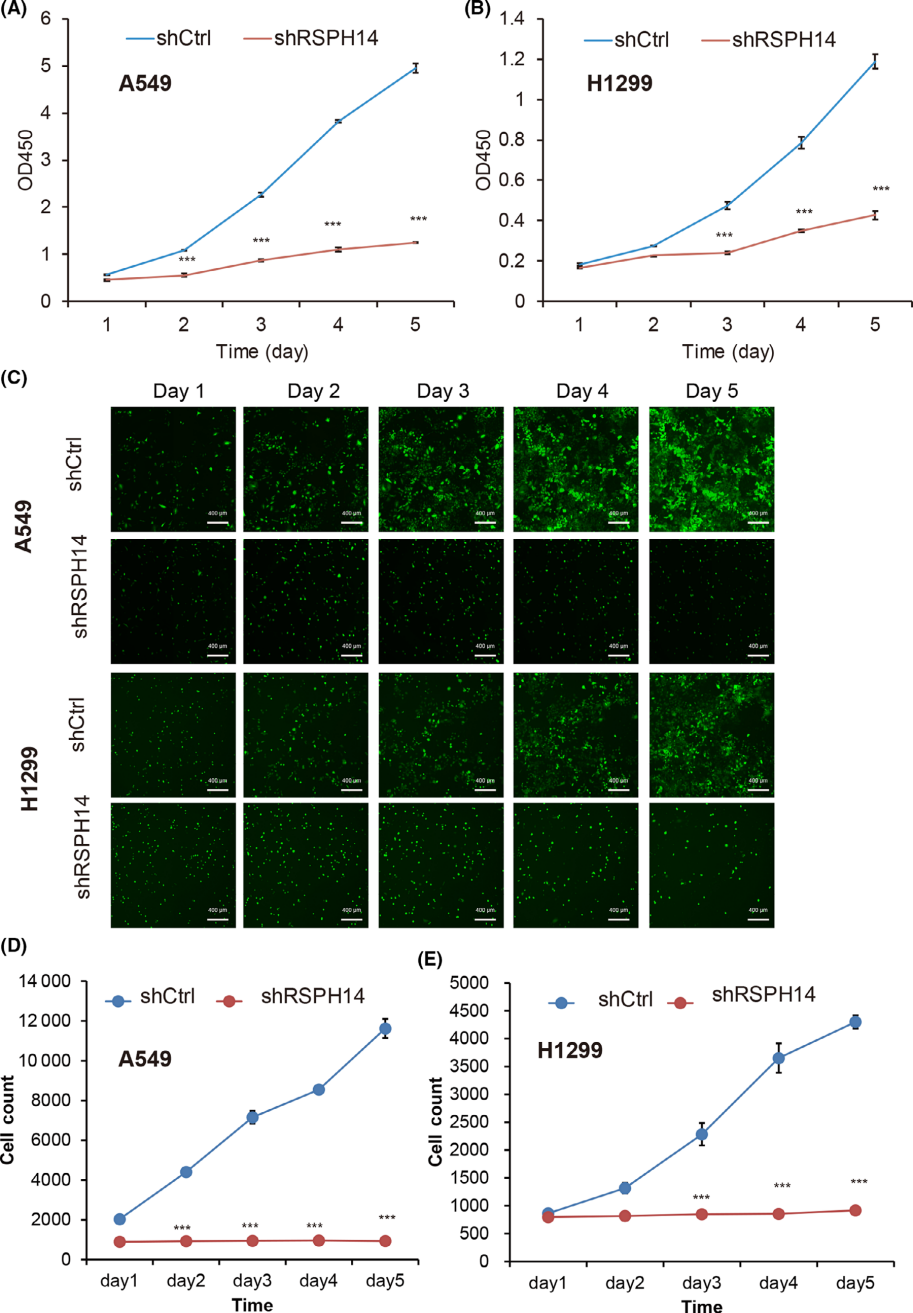
RSPH14 is correlated with cell proliferation suppression. (A–B) The proliferation of A549 and H1299 cells transfected with shRSPH14 and negative control shNC was determined using the CCK‐8 assay. (C) The proliferation of A549 and H1299 cells infected with shRSPH14 and negative control shRNA was determined using the Adherent cell cytometry system Celigo® assay. Magnification ×100. (D–E) The quantification of A549 and H1299 cell numbers after infecting with shRSPH14 and negative control shRNA. Scale bars: 400 μm. Student’s *t*‐test was used to compare differences between groups. ***P < 0.001 vs. control. All data are shown as mean ± SD of three independent experiments.

RSPH14 mRNA expression was detected in NSCLC cells. The results demonstrated that RSPH14 mRNA was highly expressed in H1975, A549, and H1299 cells relative to GAPDH RNA levels (Fig. 2A). The effect of RSPH14 on NSCLC cells was subsequently investigated. A549 and H1299 cells were infected with shRSPH14 to suppress RSPH14 protein expression (Fig. 2B‐F). The results demonstrated that cell proliferation significantly decreased in shRSPH14‐transfected A549 and H1299 cells compared with control cells (Fig. 3A and B). In addition, RSPH14 knockdown suppressed cell viability by 50 and 60% in A549 and H1299 cells, respectively, at day 3 compared with the control groups.

Celigo® analysis was performed to assess the effect of RSPH14 knockdown on NSCLC cell proliferation. As presented in Fig. 3, the number of A549 (Fig. 3C and D) and H1299 (Fig. 3C and E) cells decreased by 88.3 and 65.0%, respectively, following RSPH14 knockdown compared with the control group.

### RSPH14 knockdown inhibits cell cycle progression and induces apoptosis of NSCLC cells

Increasing evidence suggests that cancer is caused by an imbalance between cell cycle progression and apoptosis. Thus, the present study investigated whether RSPH14 modulates the cell cycle and apoptosis of NSCLC cells.

**Fig. 4:**
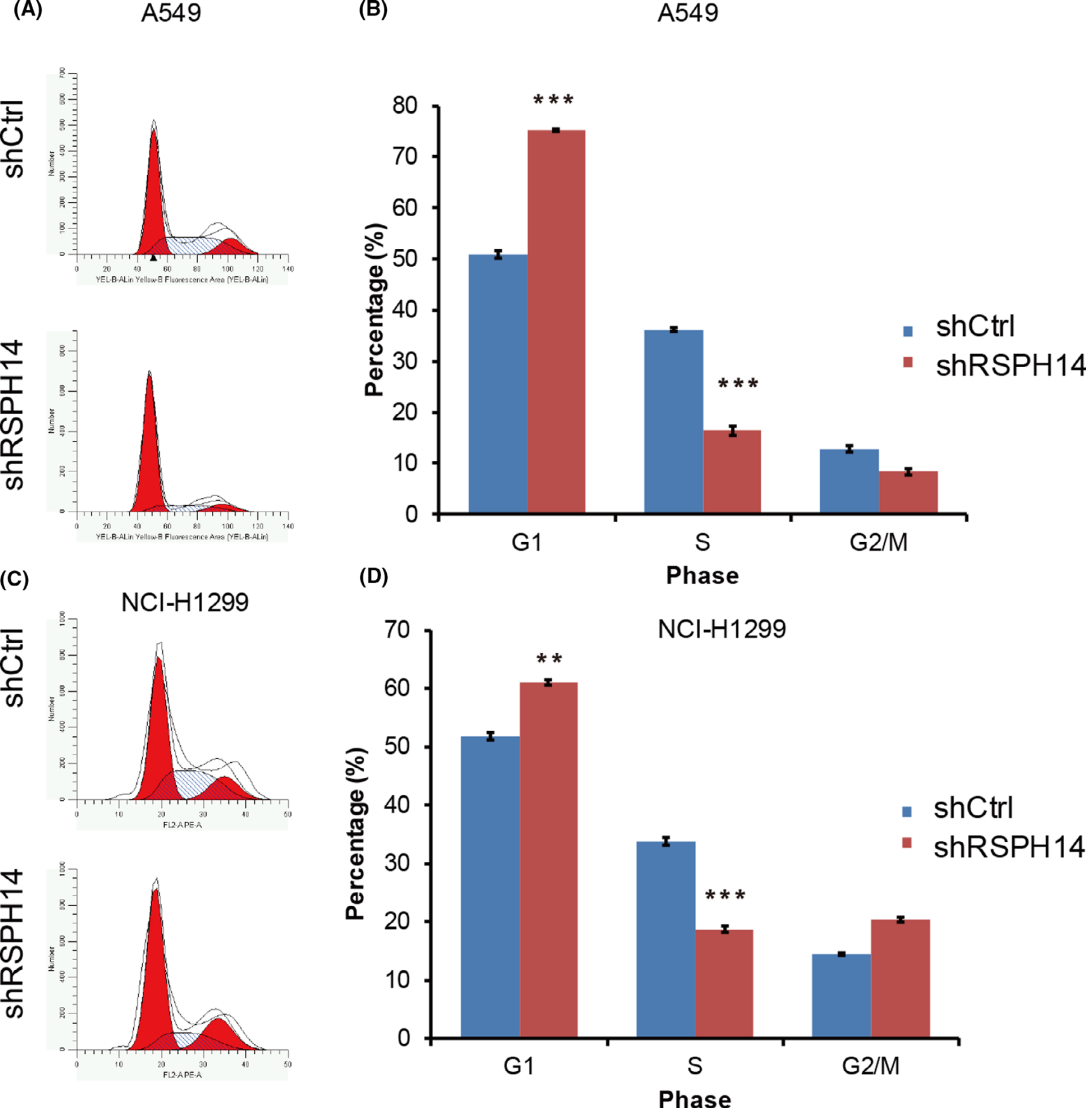
RSPH14 knockdown suppressed cell cycle progression in NSCLC. (A, C) The cell cycle of A549 (A) and H1299 (C) cells transfected with shRSPH14 and negative control shRNA was determined using the FACSanto flow cytometer assay. (B, D) A549 (B) and H1299 (D) cell cycle distribution was analyzed using a FACSanto flow cytometer (BD Biosciences) and modfit LT 3.0 software. Student’s t‐test was used to compare differences between groups. **P < 0.01, ***P < 0.001 vs. control. All data are shown as mean ± SD of three independent experiments.

Flow cytometry was performed to assess changes in the cell cycle. RSPH14 knockdown significantly increased the percentages of A549 (Fig. 4A and B) and H1299 (Fig. 4C and D) cells in the G_1_ phase, while the number of cells in the S phase notably decreased. Taken together, these results suggest that RSPH14 enhances cell cycle progression in NSCLC.

**Fig. 5:**
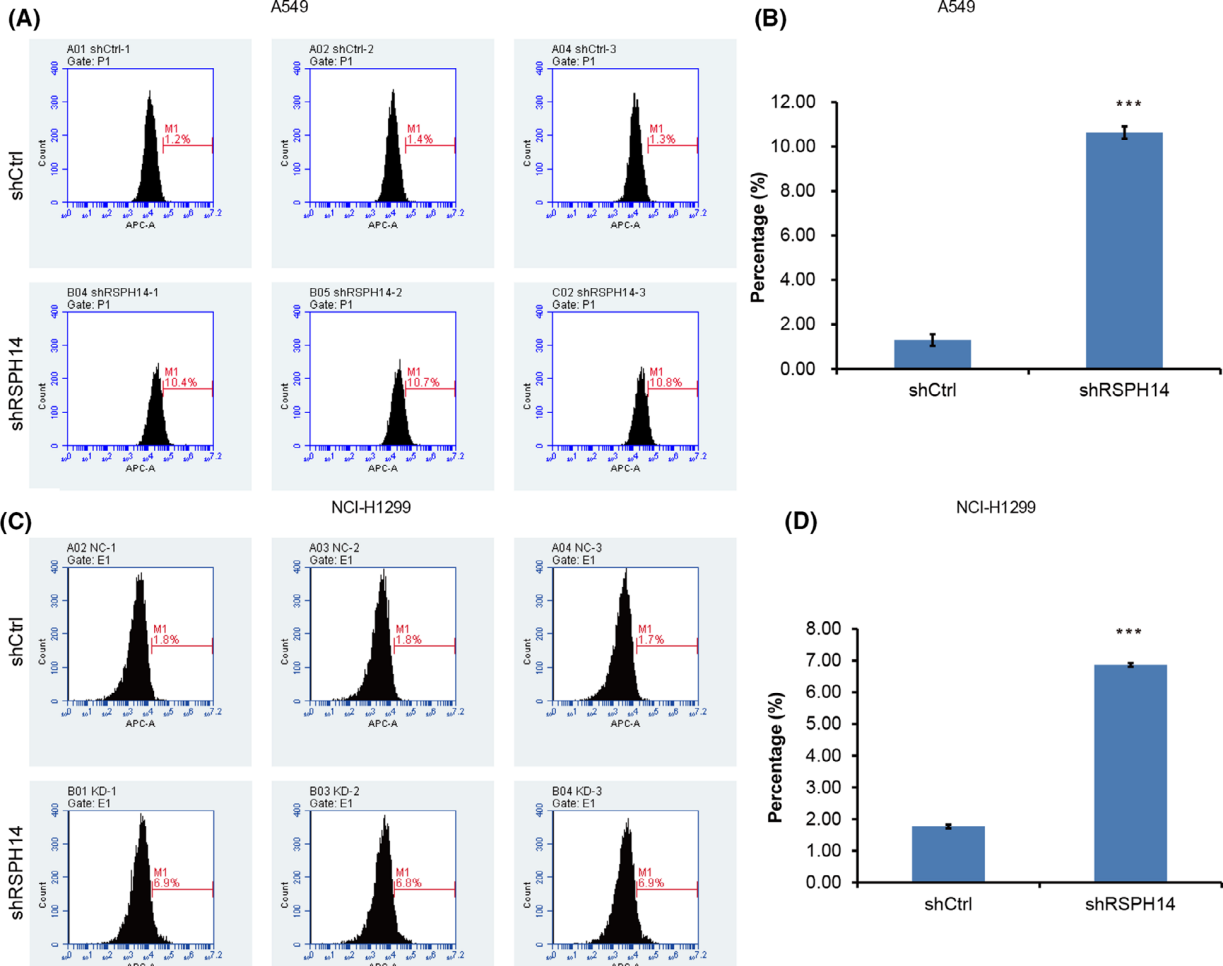
RSPH14 knockdown enhanced cell apoptosis in NSCLC. (A, C) The apoptosis of A549 (A) and H1299 (C) cells transfected with sHRSPH14 and negative control shRNA was determined using the FACSanto flow cytometer assay. (B, D) A549 (B) and H1299 (D) cell apoptosis was analyzed using a FACSanto flow cytometer (BD Biosciences) and modfit LT 3.0 software. Student’s *t*‐test was used to compare differences between groups. ***P < 0.001 vs. control. All data are shown as mean ± SD of three independent experiments.

The present study investigated whether RSPH14 is involved in promoting cell proliferation by inducing cell apoptosis. Flow cytometry combined with Annexin V‐APC single‐color staining was performed to assess the effect of RSPH14 on cell apoptosis. The results demonstrated that the number of apoptotic A549 (Fig. 5A and B) and H1299 (Fig. 5C and D) cells markedly increased following RSPH14 knockdown, which was confirmed by the significant increase of Annexin V‐APC‐positive cells. Collectively, these results suggest that RSPH14 knockdown notably enhances NSCLC cell apoptosis.

### Proteomics analysis and bioinformatics analysis of RSPH14 in NSCLC

**Fig. 6:**
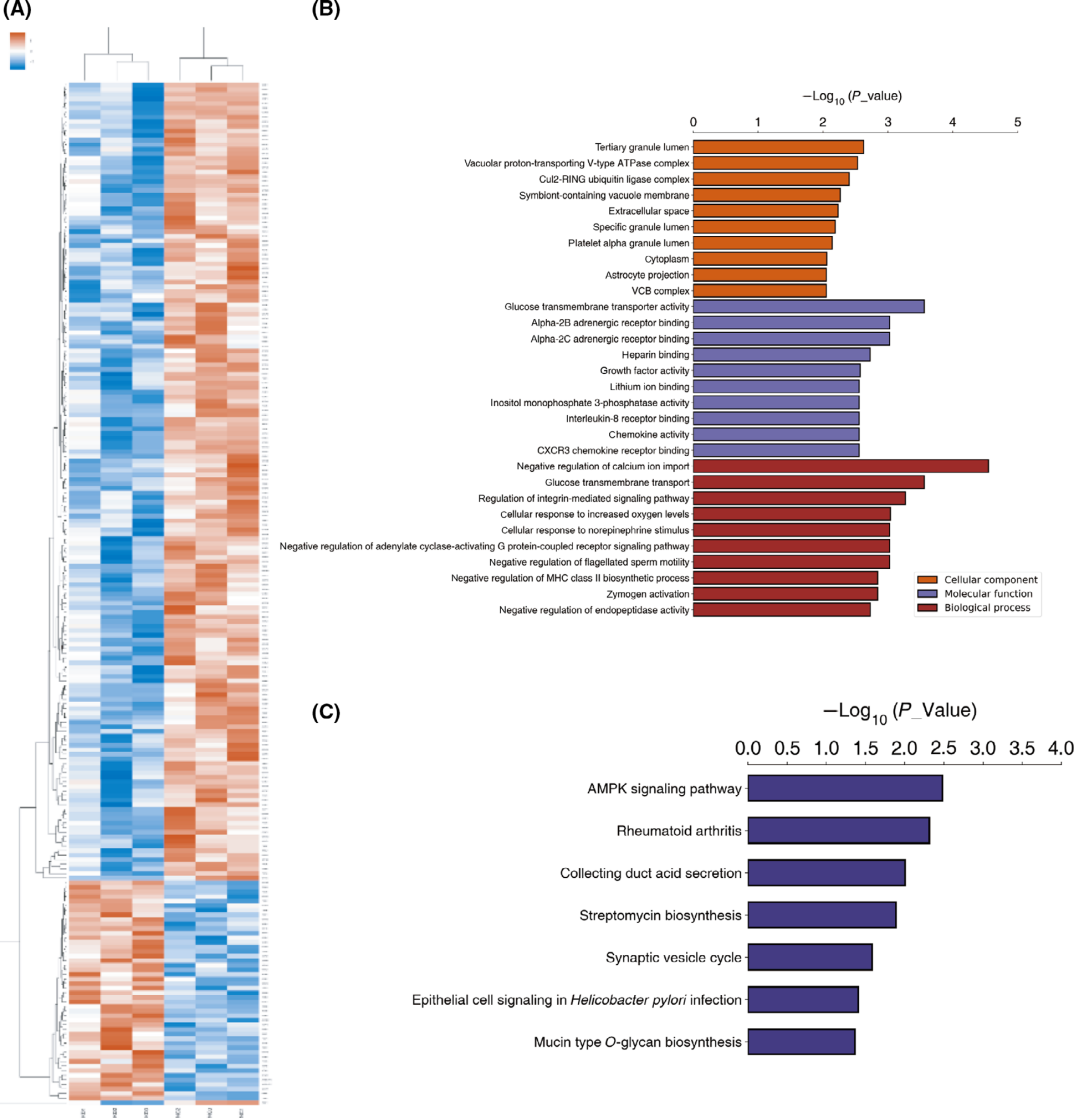
Proteomics analysis and bioinformatics analysis of RSPH14 in NSCLC. (A) Proteomics analysis was used to evaluate the change of protein levels after RSPH14 knockdown in A549 cells. (B) Gene set enrichment analyses revealed the potential functions of RSPH14 in NSCLC. (C) KEGG pathway analysis revealed the potential pathways regulated by RSPH14 in NSCLC. The Database for Annotation, Visualization and Integrated Discovery (version 6.7; http://david.abcc.ncifcrf.gov) was used to perform functional analysis. *P* < 0.05 was considered to indicate a statistically significant difference.

Given that the underlying molecular mechanisms of RSPH14 in NSCLC remain unknown, proteomics analysis was performed to assess the changes in RSPH14 protein expression following RSPH14 knockdown in A549 cells. The protein expression levels of 175 genes were downregulated, while the protein levels of 48 genes were upregulated following RSPH14 knockdown in A549 cells (Fig. 6A).

Gene set enrichment analysis demonstrated that RSPH14 targets were involved in calcium ion import regulation, glucose transmembrane transporter activity, glucose transmembrane transport, integrin‐mediated signaling pathway, alpha‐2B and alpha‐2C adrenergic receptor binding, cellular response to norepinephrine stimulus, and adenylate cyclase‐activating G protein‐coupled receptor signaling pathway, and cellular response to increased oxygen levels (Fig. 6B). KEGG pathway analysis indicated that RSPH14 targets were associated with the regulation AMPK signaling pathway, rheumatoid arthritis, collecting duct acid secretion, streptomycin biosynthesis, synaptic vesicle cycle, epithelial cell signaling in *Helicobacter pylori* infection, and mucin type O‐glycan biosynthesis (Fig. 6C).

## Discussion

NSCLC is the most common subtype of lung cancer. It is important to understand the molecular mechanisms involved in the development of NSCLC to develop effective therapies and identify novel biomarkers for NSCLC. Over the past decades, several key regulators were revealed to modulate cancer cell proliferation, cell cycle, and apoptosis in NSCLC, such as NUP37 [19], ARHGAP24 [20], and PTEN [21]. Recent studies have demonstrated that RSPH14 is associated with human diseases, such as in rhabdoid tumors, duodenal adenocarcinoma [14], congenital heart disease [15], meningiomas [16], and circulating parathyroid hormone formation [17]. However, the molecular mechanism underlying the regulatory role of RSPH14 in NSCLC tumorigenesis remains unknown.

In this study, our results demonstrated that RSPH14 expression was upregulated in NSCLC samples compared with that in normal samples. In addition, bioinformatics analysis demonstrated that high RSPH14 expression was associated with a shorter overall survival time in patients with NSCLC. Taken together, these results suggest that RSPH14 may be a potential biomarker in NSCLC. The biological functions of RSPH14 in human cancers remain largely unknown. The results of the present study suggest that RSPH14 may act as an important regulator of the progression of NSCLC. Notably, downregulated RSPH14 expression was associated with decreased cell proliferation. In addition, the results demonstrated that RSPH14 was involved in regulating cancer apoptosis and cell cycle. RSPH14 knockdown markedly promoted cell cycle arrest in the G_1_ phase and cell apoptosis compared with the control group. Collectively, these results suggest that RSPH14 acts as an oncogene in NSCLC.

Notably, as an energy sensor, AMPK has been reported to be a crucial regulator of cell apoptosis, proliferation, and autophagy [22]. AMPK modulates cell cycle arrest via multiple mechanisms. For example, AMPK can regulate p53 proteasomal turnover by phosphorylating MDMX. In addition, AMPK directly phosphorylates p53 to increase its stability and initiate cell cycle arrest [23]. AMPK is a key apoptosis regulator, which functions by modulating the regulation and stability of the p53, mTORC1, and JNK pathways [24, 25]. In NSCLC, AMPK was also reported to have a key role. For example, AMPK‐dependent phosphorylation of HDAC8 promotes lung cancer cell survival under glucose starvation via regulating PGM1 expression [26]. AMPK mediated the effect of Polyphyllin I on the growth and autophagy of NSCLC. A recent study showed AMPK‐mediated induction of lysosomal function supports NSCLC cell fitness [27]. AMPK is required to support tumor growth in murine Kras‐dependent lung cancer models [28]. However, the upstream regulators of AMPK remained largely unclear. Interestingly, the results of the present study demonstrated that RSPH14 was associated with the regulation of AMPK signaling pathway via multiple genes. Of note, proteomics and bioinformatic analyses were performed in this study to explore the underlying molecular mechanisms of RSPH14 in NSCLC. Besides AMPK signaling, multiple cancer‐related pathways were also revealed to be related to RSPH14, such as calcium ion import regulation, glucose transmembrane transporter activity, glucose transmembrane transport, collecting duct acid secretion, streptomycin biosynthesis, and synaptic vesicle cycle.

Despite this study for the confirmed that RSPH14 is a key cell cycle and apoptosis regulator in NSCLC, several limitations should be noted. Firstly, more clinical samples will be collected and used for the validation of the correlation between clinical parameters and RSPH14 expression. Secondly, the effects of RSPH14 on AMPK signaling should be confirmed using experimental assays. Thirdly, the in vivo assays should be performed to further demonstrate the effect of RSPH14 on NSCLC proliferation and apoptosis.

In conclusion, the results of the present study demonstrated that RSPH14 promoted NSCLC cell proliferation by suppressing cell cycle arrest and apoptosis. In addition, clinical analysis demonstrated that RSPH14 expression was upregulated in NSCLC samples, which was associated with a shorter overall survival time of patients with NSCLC. Proteomics analysis demonstrated that RSPH14 modulated AMPK signaling. Taken together, these results suggest that RSPH14 may be a promising biomarker for the prognosis and treatment of patients with NSCLC.

## Conflict of interest

The authors declare that they have no competing interests.

## Authors’ contributions

All authors agree to be accountable for all aspects of the research in ensuring that the accuracy or integrity of any part of the work is appropriately investigated and resolved. All authors read and approved the final manuscript.

## Data Availability

The datasets used and/or analyzed during the current study are available from the corresponding author on reasonable request.
